# Citrate Mediates Crosstalk between Mitochondria and the Nucleus to Promote Human Mesenchymal Stem Cell In Vitro Osteogenesis

**DOI:** 10.3390/cells9041034

**Published:** 2020-04-21

**Authors:** Claudia Morganti, Massimo Bonora, Saverio Marchi, Letizia Ferroni, Chiara Gardin, Mariusz R. Wieckowski, Carlotta Giorgi, Paolo Pinton, Barbara Zavan

**Affiliations:** 1 Department of Medical Sciences, University of Ferrara, 44121 Ferrara, Italy; mrgcld@unife.it (C.M.); bnrmsm1@unife.it (M.B.); mrcsvr@unife.it (S.M.); grgclt@unife.it (C.G.); 2 Laboratorio per le Tecnologie delle Terapie Avanzate (LTTA), University of Ferrara, 44121 Ferrara, Italy; 3 Maria Cecilia Hospital, GVM Care & Research, Via Corriera 1, Cotignola, 48033 Ravenna, Italy; frrltz@unife.it (L.F.); chiara.gardin@unife.it (C.G.); 4 Laboratory of Mitochondrial Biology and Metabolism, Nencki Institute of Experimental Biology, 02-093 Warsaw, Poland; m.wieckowski@nencki.gov.pl; 5 Department of Morphology, Surgery and Experimental Medicine, Section of Pathology, Oncology, University of Ferrara, 44121 Ferrara, Italy

**Keywords:** osteogenesis, citrate, α-ketoglutarate, mitochondrial metabolism, mesenchymal stem cells, mitochondria-nucleus communication

## Abstract

Citrate, generated in the mitochondria, is a key metabolite that might link metabolism with signaling, chromatin structure and transcription to orchestrate mesenchymal stem cells (MSCs) fate determination. Based on a detailed morphological analysis of 3D reconstruction of mitochondria and nuclei in single cells, we identified contact sites between these organelles that drastically increase in volume and number during the early stage of mesenchymal stem cell differentiation. These contact sites create a microdomain that facilitates exchange of signals from mitochondria to the nucleus. Interestingly, we found that the citrate derived from mitochondria is necessary for osteogenic lineage determination. Indeed, inhibition of the citrate transporter system dramatically affected osteogenesis, reduced citrate levels that could be converted in α-ketoglutarate, and consequently affected epigenetic marker H3K9me3 associated with the osteogenesis differentiation process. These findings highlight that mitochondrial metabolites play key regulatory roles in the MSCs differentiation process. Further in-depth investigation is needed to provide novel therapeutic strategies in the field of regenerative medicine.

## 1. Introduction

Mesenchymal stem cells (MSCs) are multipotent cells that give rise to osteoblasts, adipocytes, and chondrocytes [1,2,3], whereby the studying of their differentiation processes is of great interest for their promising application in tissue engineering of mesenchymal tissues.

MSC nuclear reprogramming during differentiation processes has been widely characterized [4], but several recent studies have focused on the novel mechanisms involving mitochondrial activities in stem cell biology.

Variations in the abundances, morphology, and functions of mitochondria in different stem cell types have been described [3,5,6,7,8,9], and it has been established that upregulation of mitochondrial biogenesis and metabolic shifts toward oxidative phosphorylation are hallmarks of differentiation in MSCs [10,11]. In particular, osteogenesis is followed by the development of cristae, with increased levels of proteins involved in mitochondrial biogenesis as well as enzymes of the tricarboxylic acid (TCA) cycle and subunits of respiratory chain complexes. Consequently, the oxygen consumption rate, the mitochondrial membrane potential, and the intracellular ATP content also increase, indicating enhanced oxidative metabolism in mitochondria during MSC differentiation [11,12].

In addition to a well-established role in cellular energy metabolism, mitochondria are critical mediators of cell signaling [13]; however, little is known regarding the direct effect of metabolic pathway activity on chromatin dynamics and MSC differentiation. Extensive epigenetic remodeling is necessary for the differentiation of MSCs, including covalent histone or DNA modification by methylation, acetylation, and glycosylation to regulate transcription. Moreover, recent evidence suggests that metabolic intermediates of cellular metabolism are required as cofactors for epigenetic modulators, providing a direct link between mitochondrial metabolism and gene expression [14].

In this study, we identified citrate and its derivative α-ketoglutarate (αKG) as pivotal mitochondrial metabolites that play a specific role in stem cell fate determination toward the osteogenic lineage.

## 2. Materials and Methods

### 2.1. Cell Culture

MSCs were extracted from human adipose tissues of healthy female patients undergoing cosmetic surgery procedures following guidelines from the Clinic of Plastic Surgery, University of Padova. Adipose tissues were digested with 0.075% collagenase type II from *Clostridium hystoliticum* (Sigma–Aldrich, St Louis, MO, USA) in phosphate-buffered saline (PBS). Floating adipocytes were discarded, and cells from the stromal-vascular fraction were pelleted, rinsed with medium, and centrifuged. MSCs were obtained after a red blood cell lysis step in NH_4_Cl for 10 min at room temperature.

### 2.2. Flow Cytometry

After dissociation by trypsin, cells were suspended in flow cytometry staining buffer (R&D Systems, Minneapolis, MN, USA) at a final cell concentration of 1 × 10^6^ cells/mL. After 30 min of incubation with mouse anti-human CD14 R-PE, CD34 FITC, CD44 FITC, CD45 APC, CD73 APC, CD90 R-PE, CD105 PE-Cy 7, and HLA-DR FITC (all purchased from eBioscience TM, Thermo Fisher Scientific, Waltham, MA, USA), cells were washed twice with 2 mL of flow cytometry staining buffer. The labeled cells were suspended in 500 µL of flow cytometry staining buffer, and analyzed on Attune NxT flow cytometer (Thermo Fisher Scientific).

### 2.3. In Vitro Differentiation Protocol

MSCs isolated from human adipose tissues were grown in Dulbecco’s Modified Eagle’s medium (DMEM)-low-glucose (LG) (EuroClone S.p.A., Milan, Italy) supplemented with 10% fetal bovine serum, 2 mM L-glutamine, and antibiotics (penicillin 100 μg/mL and streptomycin 10 μg/mL) at 37 °C in a humidified atmosphere of 5% CO_2_.

For adipogenic differentiation, DMEM-LG was replaced with DMEM-high-glucose (HG) (EuroClone S.p.A) plus 10 µg/mL insulin, 0.5 mM IBMX, 0.1 mM indomethacin, and 1 µM dexamethasone for 3, 7, and 21 days. For osteogenic differentiation, LG was replaced with DMEM-HG plus 10 nM dexamethasone, 10 ng/mL FGF-β, and 10 mM β-glycerophosphate for 3, 7, and 21 days. All growth factors were purchased from Sigma-Aldrich.

### 2.4. Immunofluorescence Microscopy

Immunofluorescence microscopy was performed according to standard procedures. Briefly, cells were fixed in 4% PFA for 20 min at room temperature, washed three times with PBS and permeabilized with 0.1% Triton X-100 for 5 min at room temperature. Thereafter, unspecific binding sites were blocked by incubating cells in PBS supplemented with 2% bovine serum albumin (BSA, used as blocking buffer) for 1 h at room temperature. Cells were then incubated overnight at 4 °C with primary antibodies and then revealed using appropriate AlexaFluor 488^®^ or AlexaFluor 594^®^ conjugates (Thermo Fischer Scientific). The nucleus was stained by Hoechst. Images were acquired using an LSM 510 confocal microscope (Carl Zeiss Microscopy, LLC, Jena, Germany) with a Plan-Apochromat 63x/1.4 oil objective (Carl Zeiss Microscopy, LLC).

### 2.5. Measurement of Mitochondrial Number, Volume, and Identification of Mitochondrion-Nucleus Contact Sites

After differentiation, cells were fixed on a glass coverslip; nuclei were stained with Hoechst and mitochondria with an anti-TOM20 antibody (Ab). After Z-stack acquisition, images were deconvoluted using Huygens Essential software (Scientific Volume Imaging B.V., Hilversum, The Netherlands), and a 3D reconstruction of the mitochondrial network and nucleus in a single cell was created using Imaris 7 (Bitplan, Zurich, Switzerland) software.

The mitochondrial number and volume were measured for single cell. The TOM20 channel was used to create the 3D mitochondrial isosurface by Imaris 7, and the total volume and number of objects were analyzed for each of these isosurfaces.

The colocalization between TOM20 and Hoechst, the two fluorescent signals were analyzed by the Imaris colocalization tool, and a colocalization channel was created. Finally, two isosurfaces (mitochondria and colocalization channel) were generated, and the total volume and number of objects were analyzed for each of these isosurfaces.

### 2.6. Antibodies

The following primary antibodies were used for immunoblotting: rabbit anti-GAPDH [2118] (1:5000) from Cell Signaling; rabbit anti-TOM20 [sc-11415] (1:1000) and mouse anti-HSP60 [sc-13115] (1:1000) from Santa Cruz Biotechnology (Dallas, TX, USA); anti-VDAC [ab-15895] (1:1000) from Abcam (Cambridge, UK); anti-TIM23 [611222] (1:1000) from BD Bioscience (San Jose, CA, USA). The following primary antibodies were used for immunofluorescence images: TOM20 [sc-11415] (1:100) from Santa Cruz Biotechnology, H3 [14269] (1:100), H3K9ac [9649] (1:100), and H3K9me3 [13969] (1:100) from Cell Signaling (Danvers, MA, USA).

### 2.7. XF Bioenergetic Analysis

Oxygen-consumption rates were measured using the SeahorseXF96 instrument according to the manufacturer’s protocols. After differentiation, MSCs were seeded in a poly-lysine-coated XF96 microplate at a density of 50,000 cells per well in 175 μL unbuffered XF assay medium (pH 7.4) supplemented with 5.5 mM glucose, 1 mM sodium pyruvate, and 1 mM glutamine for 60 min in a 37 °C non-CO_2_ incubator; sensor cartridges were calibrated prior to the start of assays. Respiration was measured in four blocks of three for 3 min. The first block measured the basal respiration rate. Next, 1 μM oligomycin was added to inhibit complex V (second block); 1 μM carbonylcyanide 4-(trifluoromethoxy)-phenylhydrazone (FCCP) was added to uncouple respiration (third block). Finally, 2 μM antimycin A and 2 mM rotenone were added to inhibit complex III (fourth block). All reagents were from Agilent Technologies, Seahorse Biosciences (Santa Clara, CA, USA). Immediately after finishing the measurements, cells were washed with PBS, fixed with 4% paraformaldehyde (PFA), and stained with 0.1% crystal violet (1 mol/L acetic acid); absorbance at 595 nm was measured as an index of the number of cells.

### 2.8. Oil Red O Staining

Oil red O (Sigma-Aldrich) staining of cytoplasmic droplets of neutral lipids was performed according to a standard procedure. Monolayer cultures were fixed in 10% neutral-buffered formalin for 10 min. Oil Red O working solution was added to wells for 10 min at room temperature. After washing, stained cells were assessed by light microscopy.

### 2.9. Alizarin Red Staining

For Alizarin Red S (ARS) staining, cells were fixed with 10% neutral-buffered formalin for 10 min and stained with 40 mM Alizarin Red S solution (pH 4.2) at room temperature for 10 min. After washing, the stained cells were examined by light microscopy.

### 2.10. RNA Extraction and Quantitative Reverse Transcription Polymerase Chain Reaction (RT-PCR)

Total RNA was extracted with TRIzol Reagent (Invitrogen, Carlsbad, CA, USA). RNA was purified with RNeasy Mini Kit (QiagenGmbH, Hilden, Germany), and DNase digestion was performed with RNase-Free DNase Set (Qiagen). The RNA quality and concentration were measured using a NanoDrop TM ND-1000 (Thermo Scientific). For first-strand cDNA synthesis, 1000 ng of total RNA from each sample was reverse-transcribed with M-MLV Reverse Transcriptase (Invitrogen/ Thermo Fisher Scientific, Waltham, MA, USA) following the manufacturer’s protocol.

Real-time PCR was carried out using primers designed for human genes at a concentration of 300 nM and with FastStart SYBR GreenMaster (Roche Diagnostics, Mannheim, Germany) and a Rotor-Gene 3000 (Corbett Research, Sydney, Australia). The thermal cycling conditions were as follows: 10-min denaturation at 95 °C, followed by 40 cycles of denaturation for 10 s at 95 °C, annealing for 20 s at 60 °C, and elongation for 30 s at 72 °C. Data analysis was performed using the 2ΔΔCt method: values were normalized to expression of the Transferrin Receptor (TFRC) internal reference, and results are reported as the fold change of target genes in the test group with respect to the control.

### 2.11. Cell Growth Assay

After 21 days of treatment, cells were washed with PBS, fixed in 4% PFA, and stained with 0.1% crystal violet. Crystal violet was dissolved with 1 mol/L acetic acid, and absorbance at 595 nm was measured.

### 2.12. Statistical Analysis

Results are expressed as the mean ± SD. The probability of significant differences between experimental groups was determined by analysis of variance (ANOVA), and results from treatments showing significant overall changes were subjected to post-hoc Bonferroni tests. Student’s *t*-test was employed to determine statistical significance between two groups. *p* values < 0.05 were considered statistically significant. Different labels indicate * *p* < 0.05, ** *p* < 0.001, *** *p* < 0.0001, and **** *p*< 0.00001.

## 3. Results

### 3.1. Mitochondrion-Nucleus Contact Sites Increase Following Differentiation Processes and Mitochondrial Modification

Stem cell fate regulation is principally characterized by marked nuclear reprogramming, yet the important dynamic rearrangement of mitochondria during differentiation suggests their direct involvement in these finely tuned processes.

To investigate the role of mitochondria in MSCs, cells were isolated from human adipose tissues and characterized for surface markers (Supplementary Figure S1) as previously described [15,16]. Adipose-derived MSCs were then differentiated into adipogenic (AD) and osteogenic (OS) lineages for 21 days.

**Figure 1:**
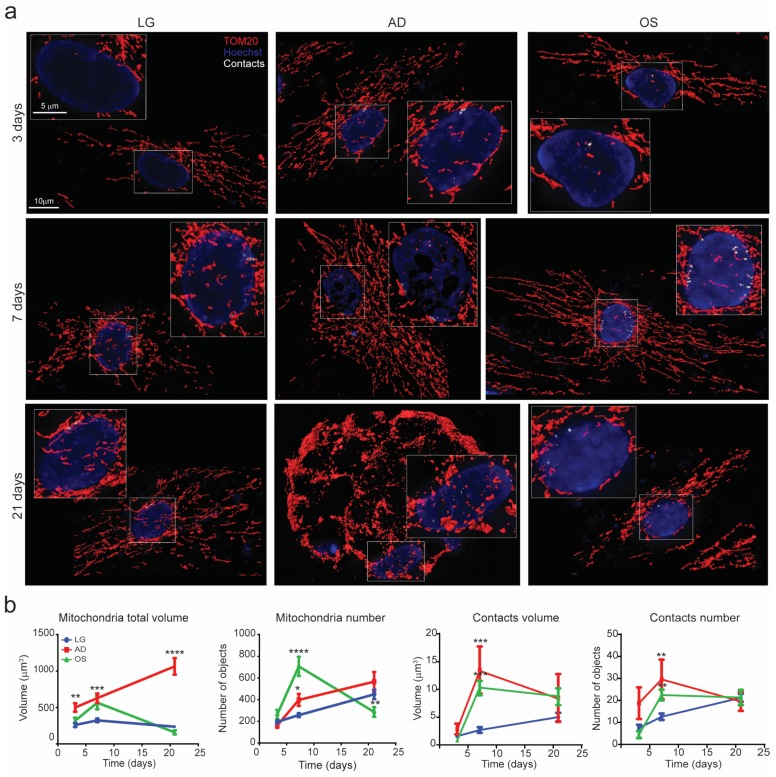
Mitochondria-nucleus contacts. Mesenchymal stem cells (MSCs) were cultured in low-glucose (LG), adipogenic (AD) and osteogenic (OS) media for 3, 7, and 21 days, as indicated. (**a**) Representative 3D images of mitochondrial morphology, as detected by anti-TOM20 Ab immunofluorescence, and contact sites identified by creating the isosurface of the colocalization channel between nuclear staining (Hoechst) and TOM20. Magnification 40x. Scale bar 10 μm (in zoom panel, scale bar 5 μm). (**b**) Quantification of mitochondrial total volume, number, and total volume and number of contacts. Data are derived from ≥45 acquired cells/condition from ≥3 independent experiments. Data are shown as the mean ± SD. ANOVA, * *p* < 0.1, ** *p* < 0.01, *** *p* < 0.001, **** *p* < 0.0001 with respect to the LG condition at the same time point.

Mitochondria were stained by Ab-TOM20 and nuclei by Hoechst, and the colocalization channel between the two signals was used to identify sites of contact between the two organelles in a single cell. Using 3D reconstruction of mitochondria and the colocalization channel, we showed that the mitochondrial total volume and number, as well as the volume and number of mitochondrion-nucleus contacts, increased dramatically after 7 days of adipogenic and osteogenic differentiation (Figure 1a,b).

**Figure 2:**
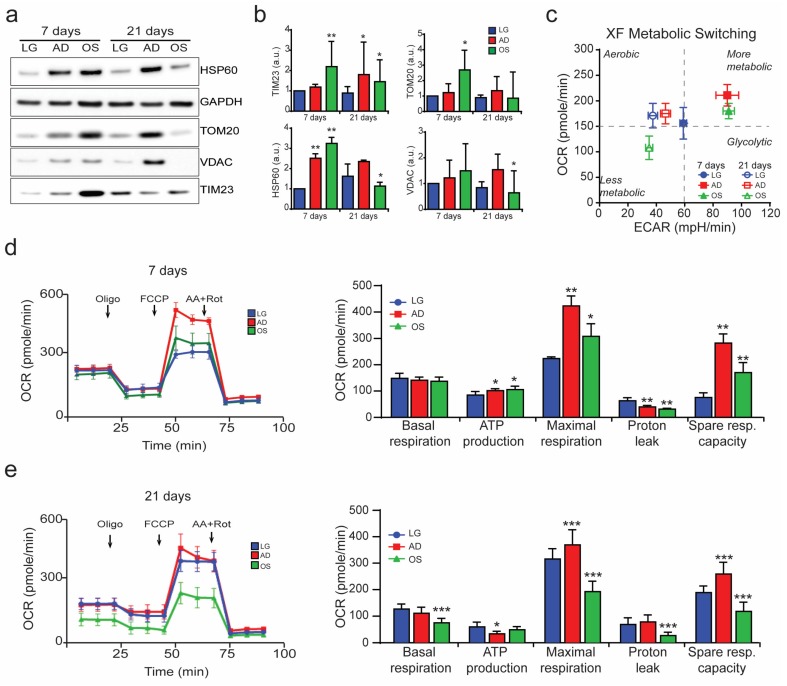
Mitochondria in MSC differentiation processes. (**a**) Representative immunoblot and (**b**) quantification of HSP60, TOM20, VDAC, and TIM23 protein levels normalized to GAPDH levels. (**c**) XF phenograms representing the metabolic switching of MSCs during differentiation, as detected using an Extracellular Flux Analyzer (Seahorse Bioscience). (**d**,**e**) Oxygen consumption rate (OCR) measurements and relative derived parameters after the addition of oligomycin [1 µM], 1 μM carbonylcyanide 4-(trifluoromethoxy)-phenylhydrazone (FCCP) [1 µM], and antimycin A [2 µM] + rotenone [2 µM] in MSCs cultured in low-glucose (LG), adipogenic (AD), and osteogenic (OS) media for (**d**) 7 and (**e**) 21 days. Data are derived from ≥45 acquired cells/condition from ≥3 independent experiments and are shown as the mean ± SD. ANOVA, * *p* < 0.1, ** *p* < 0.01, *** *p* < 0.001, **** *p* < 0.0001 with respect to the LG condition at the same time point.

In addition, alterations in mitochondrial content and physiology were analyzed via detection of mitochondrial markers by immunoblotting and using a Seahorse Extracellular Flux Analyzer (Agilent), which measures the mitochondrial oxygen consumption rate (OCR) and the extracellular acidification rate (ECAR), key metrics of mitochondrial function. Our data revealed that adipogenesis is characterized by a progressive increase in mitochondrial mass, as shown by increase in levels of mitochondrial proteins such as HSP60, TOM20, VDAC, and TIM23 (Figure 2a,b). In addition, analysis of the OCR/ECAR phenogram confirmed that differentiation processes are characterized by an essential metabolic switch. Indeed, differentiating MSCs switched from glycolytic to oxidative metabolism, as demonstrated by a reduction in ECAR (Figure 2c,d). In contrast to osteogenesis, two conditions were identified: after 7 days, mitochondrial mass increased drastically, particularly due to the consistent increase in the number of mitochondria; after 21 days of differentiation, mitochondrial mass decreased significantly (Figure 1a,b and Figure 2a,b), as also reflected in the metabolic phenogram (Figure 2c–e).

The important decrease in mitochondrial mass and basal respiration after 21 days of osteogenesis suggests that the mitochondria formed at 7 days should be exhausted and destined for turnover. Mitophagy is a specialized type of autophagy specific for mitochondrial degradation through lysosomes [17], and to better characterize this interesting dynamic that occurs in mitochondria during osteogenesis, levels of specific mitochondrial proteins and markers of autophagy/mitophagy were followed during osteogenesis (Supplementary Figure S2).

We showed that a decrease in mitochondrial protein levels (Supplementary Figure S2a,b) is followed by an increase in autophagy, as shown by the marker LC3-II [18] and a decrease in PARKIN [19] (Supplementary Figure S2a). Moreover, colocalization of LC3-GFP dots and TOM20 confirmed that the late phase of osteogenesis is linked to the mitophagy process (Supplementary Figure S2c).

### 3.2. Inhibition of Citrate Transporters Impairs Osteogenesis

**Figure 3:**
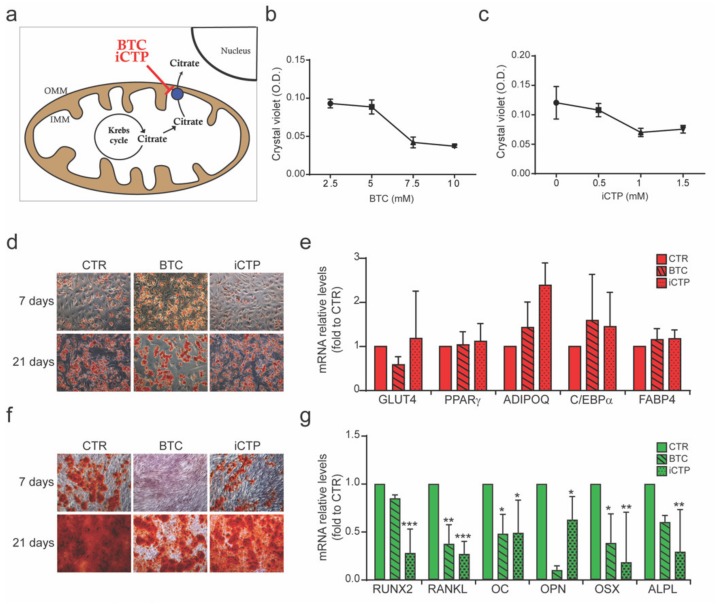
Inhibition of the citrate transport system impaired osteogenic, but not adipogenic, differentiation. MSCs were cultured in low-glucose (LG), adipogenic (AD), and osteogenic (OS) media; where indicated, 1,2,3-benzenetricarboxylate (BTC) [5 mM] and CTP inhibitor (iCTP) [500 µM] were added to inhibit the transport of citrate produced in mitochondria to the cytosol. (**a**) Schematic representation of the acetyl-CoA transport system. In the mitochondrial matrix, acetyl-CoA is converted to citrate by citrate synthase. Citrate is then transported by the citrate transporter to the cytosol, where it is reconverted to acetyl-CoA by citrate lyase. (**b**,**c**) Cell survival assay to determine the concentration of BTC (**b**) and iCTP (**c**). Adipogenic differentiation was detected by (**d**) Oil Red O staining after 7 and 21 days of differentiation and by (**e**) analysis of relative mRNA levels of adipogenic markers (GLUT4, PPARγ, ADIPOQ, C/EBPα, and FABP4) after 21 days. ANOVA-test refers to the control (CTR) condition (adipogenic medium). Osteogenic differentiation was detected by (**f**) Alizarin Red staining after 7 and 21 days of differentiation and by (**g**) analysis of relative mRNA levels of osteogenic markers (RUNX2, RANKL, osteocalcin (OC), osteopontin (OPN), osterix (OSX), and alkaline phosphatase (ALP)) after 21 days. Data are derived from ≥3 independent experiments and are shown as the mean ± SD. ANOVA-test refers to the CTR condition (osteogenic medium). * *p* < 0.5, ** *p* < 0.01, *** *p* < 0.001.

Mitochondrial metabolism plays a central role in cell energy metabolism, and the generation of mitochondrial metabolites represents a possible way by which mitochondria regulate stem cell fate. In fact, pivotal enzymes that regulate chromatin, and thus transcription, rely on mitochondrial metabolic intermediates [20,21,22]. Among the several metabolites of the TCA cycle, acetyl coenzyme A (acetyl-CoA) and αKG participate in the regulation of histone acetylation and methylation, respectively. Their common precursor is citrate, which is produced in the mitochondrial matrix and can enter the oxidative phase of the Krebs cycle or be transported from mitochondria to the cytosol where it can be reconverted in acetyl-CoA or αKG (through isocitrate as an intermediate). Citrate exits the mitochondria through mitochondrial citrate transport protein (CTP), a membrane-embedded protein that catalyzes translocation of citrate across the mitochondrial inner membrane [23] (Figure 3a).

CTP inhibitor (referred to herein as iCTP), the first purely competitive inhibitor to be discovered, inhibits of mitochondrial CTP and is more potent than 1,2,3-benzenetricarboxylate (BTC), the classic and defining inhibitor of mitochondrial CTP [24].

In the present study, MSCs were differentiated toward adipogenic and osteogenic lineages, and 5 mM BTC or 500 µM iCTP, a dose that does not affect cell proliferation, was added as indicated (Figure 3b,c). BTC and iCTP treatment did not affect MSC adipogenesis, as shown by staining of lipid drop accumulation (Figure 3d and by the mRNA levels of adipogenic markers (GLUT4, PPARγ, ADIPOQ, C/EBPα, and FABP4) (Figure 3e).

In contrast, BTC and especially iCTP strongly inhibited osteogenic differentiation. The calcium deposits detected by Alizarin Red staining were reduced in MSCs cultured in osteogenic medium plus BTC or iCTP compared to CTR conditions at both 7 and 21 days (Figure 3f). In addition, analysis of the mRNA profile of osteogenic markers (RUNX2, RANKL, osteocalcin, osteopontin, osterix, and alkaline phosphatase) confirmed that inhibition of mitochondrial citrate transport strongly impaired MSC osteogenic differentiation (Figure 3g).

**Figure 4:**
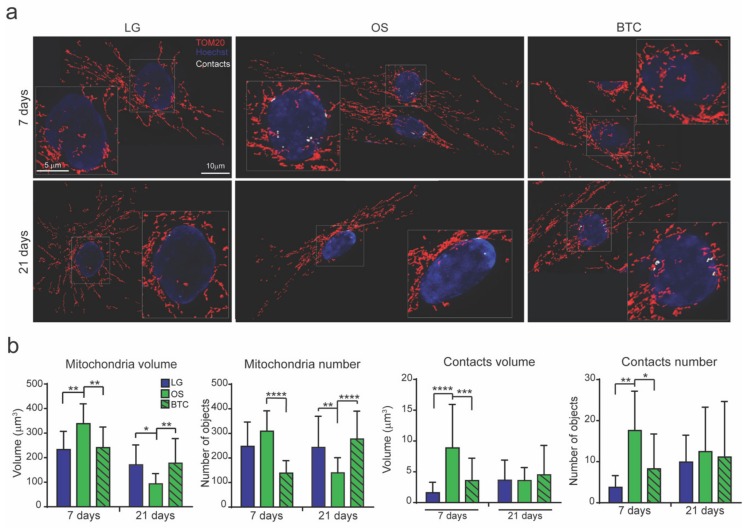
Inhibition of the citrate transport system impaired mitochondrial behavior. MSCs were cultured in low-glucose (LG), adipogenic (AD), and osteogenic (OS) media; where indicated, BTC [5 mM] and iCTP [500 µM] were added to inhibit the transport of citrate produced in mitochondria to the cytosol. (**a**) Representative images and (**b**) analysis of mitochondrial total volume and number detected by the anti-TOM20 Ab and the total volume and number of mitochondrion-nucleus contact sites. Magnification 40x. Scale bar 10 μm (in zoom panel, scale bar 5 μm). Data are derived from ≥45 acquired cells/condition from ≥3 independent experiments and are shown as the mean ± SD. ANOVA-test. * *p* < 0.5, ** *p* < 0.01, *** *p* < 0.001, **** *p* < 0.0001.

BTC also affected osteogenic-induced changes in mitochondrial morphology and mitochondrion-nucleus contact sites. As a result, MSCs treated with BTC resembled MSCs in the undifferentiated condition, confirming that mitochondrial changes are linked to the osteogenic differentiation (Figure 4a,b).

### 3.3. Citrate is Converted to αKG to Promote Osteogenesis

**Figure 5:**
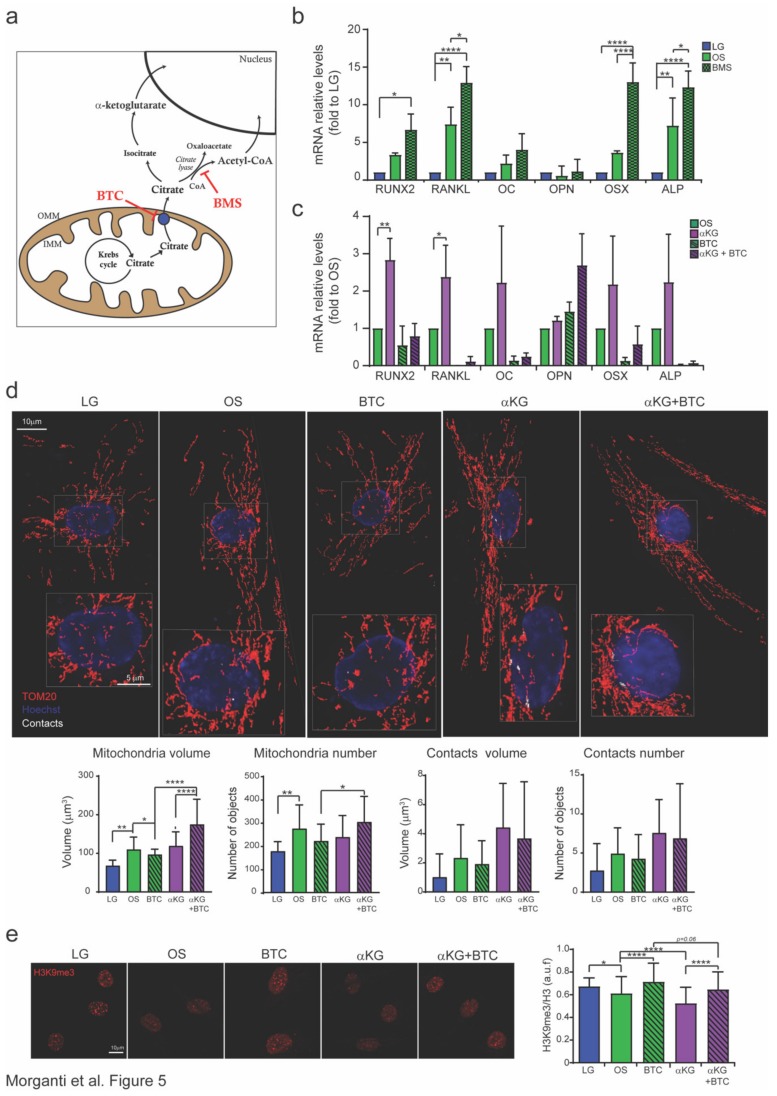
Citrate from mitochondria increases the level of α-ketoglutarate to promote osteogenesis. (**a**) Citrate can be converted to acetyl-CoA by citrate lyase (inhibited by BMS) or to α-ketoglutarate (αKG), two metabolites that can mediate mitochondrion-nucleus communication. (**b**,**c**) Analysis of relative mRNA levels of osteogenic markers (RUNX2, RANKL, osteocalcin (OC), osteopontin (OPN), osterix (OSX), and alkaline phosphatase (ALP)) in MSCs cultured in low-glucose (LG) and osteogenic media (OS); where indicated, BMS [1 mM], BTC [5 mM], and αKG [1 mM] were added for 21 days. (**d**) Representative images and analysis of mitochondrial total volume and number detected by the anti-TOM20 Ab and the total volume and number of mitochondrion-nucleus contact sites in MSCs cultured in LG and osteogenic media (OS); where indicated, BTC [5 mM] and αKG [1 mM] were added for 21 days. (**e**) Representative images and quantification of H3K9me3 in MSCs cultured in LG and OS media; where indicated, BTC [5 mM] and αKG [1 mM] were added for 21 days. Magnification 40x. Scale bar 10 μm (in zoom 5 μm). Data are derived from ≥45 acquired cells/condition from ≥3 independent experiments and are shown as the mean ± SD. ANOVA-test. * *p* < 0.5, ** *p* < 0.01, *** *p* < 0.001.

Citrate in the cytosol can be converted to acetyl-coA or αKG (Figure 5a), two key metabolites able to regulate histone acetylation and methylation states.

Several reviews have reported the effect of epigenetic modifications on MSCs multipotency and differentiation [25,26,27], among which H3K9ac and H3K9me3 are considered key epigenetic signatures indicative of transcriptional activation and inactivation, respectively [28]. These signatures interact with the osteogenic master transcriptional factor RUNX2 during osteogenesis [29].

We confirmed in our model that H3K9ac levels increased during osteogenesis (Supplementary Figure S3a) and that the osteogenesis defect caused by CTP inhibition resulted in H3K9ac levels that were comparable those of undifferentiated cells (low glucose). Without affecting cell growth, citrate conversion to acetyl-CoA by citrate lyase was inhibited by 1 mM BMS (Supplementary Figure S3c). Although BMS treatment was able to decrease the levels of H3K9ac (Supplementary Figure S3b), it also increased the osteogenic potential of MSCs (Figure 5b). This suggests that citrate accumulation due to citrate lyase inactivation promoted osteogenesis. To investigate whether αKG is necessary for osteogenesis, exogenous αKG (1 mM, Supplementary Figure S3d) was added to the differentiation medium, which promoted osteogenesis and partially rescued the phenotype due to CTP inhibition (Figure 5c).

αKG is a substrate for the Jumonji-C (JmjC) domain-containing histone demethylase family of proteins that catalyze histone demethylation [30]. Because removal of H3K9me3 marks is required for osteogenesis [31], H3K9me3 levels were investigated. We confirmed that osteogenesis is linked to a reduction in H3K9me3 marks, whereas CTP inhibition resulted in levels comparable to those of undifferentiated cells; furthermore, αKG addition promoted demethylation (Figure 5e).

Finally, to confirm that citrate should exit mitochondria and enter the nucleus to be converted into αKG and play a regulatory role in the methylation status of histones, we added αKG under conditions in which CTP was inhibited (αKG + BTC). Under this condition, osteogenic potential (Figure 5c) and methylation status (Figure 5e) were partially restored, as were mitochondrial morphology and contact sites (Figure 5d), confirming our hypothesis that citrate derived from mitochondria is essential to induce osteogenic differentiation.

## 4. Discussion

MSC differentiation has largely been investigated from a transcription factor point of view, whereas little is known concerning how metabolism might regulate this complex process. Due to the very distinct metabolic and energetic demands of differentiated cell types, stem cell differentiation requires variations in the number, structure, function, and intracellular distribution of mitochondria [32,33,34]. In particular, mitochondrial DNA copy number and intracellular ATP content as well as levels of protein subunits of the respiratory enzymes increase during osteogenic differentiation of MSCs [11,35].

In this study, we performed a detailed morphological analysis of the mitochondrial network by 3D reconstruction in a single cell. We showed that during the early phase of adipogenic and osteogenic differentiation, mitochondria volume and number increased dramatically in agreement with the enhanced mitochondrial respiration (Figure 1 and Figure 2), confirming previous evidence detected by the classical immunofluorescence technique [6,36]. The large number of mitochondria suggests increased mitochondrial biogenesis during both adipogenesis and osteogenesis. Our data are supported by a previous study [7], in which it was shown that the protein level of TOM20 was greatly increased during adipogenic differentiation, confirming the increase in mitochondrial mass. Interestingly, in the late phase of the differentiation process, adipogenic cells exhibited a conspicuous number of mitochondria, whereas osteogenic cells had few of these organelles (Figure 1 and Figure 2). Recent studies have demonstrated a pivotal role of autophagy in the osteo-differentiation of hBMSCs [37,38,39], and mitophagy has been showed to be essential for the maintenance of hematopoietic stem cell [40]. Our data showed that autophagy and mitophagy levels are dynamically regulated during osteogenesis (Supplementary Figure S2), suggesting a possible crucial role of mitophagy in the MSCs differentiation process which should be deeply investigated in future studies.

In addition to the general mitochondrial alterations commonly accepted, we deeply investigated the localization of mitochondria in relation to the nucleus, identifying sites of contact between the two organelles. It is generally accepted that mitochondria mainly accumulate around the nucleus in undifferentiated cells, whereas they are more uniformly distributed throughout the cytoplasm of differentiated cells [41,42]. Nonetheless, the number and volume of contact sites between the nucleus and mitochondria were found to increase following the early stage of the differentiation process (Figure 1). This let us to hypothesize a crucial communication between mitochondria and the nucleus, whereby contact sites create microdomains in which exchange of metabolites may be heightened to promote differentiation.

Mitochondrial metabolism might be pivotal for stem cell fate regulation; indeed, metabolites generated in the TCA cycle can act as cofactors or substrates for chromatin-modifying enzymes. In fact, a growing body of evidence suggests that the metabolic profile of cells can influence cytoplasmic signaling and epigenetics [43,44,45]. Interestingly, several mitochondrial enzymes associated with the TCA cycle are essential for epigenetic remodeling and are transiently and partially localized to the nucleus during embryonic development [46].

Citrate plays a central role in highly proliferating undifferentiated stem cells and in their differentiation toward specific functional cell types [47]. In particular, we show that inhibition of the citrate transporter system dramatically affects osteogenesis, as opposed to adipogenesis (Figure 3). This finding highlights that mitochondrial citrate is fundamental for determining stem cell fate.

Bone contains extremely high levels of citrate, which is an essential component of the apatite nanocrystal [48], and osteoblasts are the source of citrate that is incorporated into bone during bone formation [49]. Moreover, citrate functions as a chelating agent and binds physiologically relevant cations such as Ca^2+^, Zn^2+^, and Mg^2+^; thus, citrate constitutes the major store of calcium in bones [50]. For this reason, altered citrate metabolism may occur during the differentiation of mesenchymal stem cells to functional citrate-producing osteoblasts.

In addition, citrate plays a role in the regulation of epigenetic markers. Although citrate can be converted to acetyl-coA or αKG (via isocitrate), we demonstrate that conversion of citrate to the latter rather than the former allows osteogenic differentiation (Figure 5).

αKG can enter the nucleus where it is used as a substrate by ten-eleven translocation (TET) proteins for DNA demethylation and by Jumonji C domain demethylases (JHDMs) for histone demethylation (linked to context-dependent gene silencing or activation) [51].

It has been reported that the maintenance of proper α-KG levels is fundamental in determining the identity and fate of embryonic stem cells (ESCs). Specifically, a high α-KG/succinate ratio promotes the activity of DNA and histone demethylases, and modifying this ratio is sufficient to regulate multiple chromatin modifications [52]. Lastly, we linked α-KG levels to H3K9me3, an epigenetic marker associated with the osteogenesis differentiation process.

Taken together, our data suggest that during MSC differentiation, mitochondria assemble close to the nucleus to create microdomains to facilitate the exchange of metabolites from the mitochondrial matrix to the nucleus. Citrate exiting from mitochondria is converted to αKG, which promotes histone modification, leading to transcriptional activation of osteogenic-related genes. These findings highlight that mitochondrial metabolites play a key regulatory role in the differentiation process, which should be deeply investigated in the future and may reveal novel therapeutic strategies in the field of regenerative medicine.

## Supplementary Materials

The following are available online at https://www.mdpi.com/2073-4409/9/4/1034/s1, Figure S1: Detection of cell surface markers by flow cytometry, Figure S2: Mitophagy activation in the late stage of osteogenesis, Figure S3: Modulation of citrate transport system.
