# Supplementary material for: Citrate Mediates Crosstalk between Mitochondria and the Nucleus to Promote Human Mesenchymal Stem Cell In Vitro Osteogenesis
Source: Cells. 2020 Apr 21;9(4):1034. doi: 10.3390/cells9041034 (PMC7226543; doi:10.3390/cells9041034)
Supplement: Supplementary file 1 [file cells-09-01034-s001.pdf]

cells

Citrate Mediates Crosstalk between Mitochondria and the Nucleus to Promote Human Mesenchymal Stem Cell in vitro Osteogenesis

Claudia Morganti [1,2], Massimo Bonora [1,2], Saverio Marchi [1,2], Letizia Ferroni [3], Chiara Gardin [3], Mariusz R. Wieckowski [4], Carlotta Giorgi [1,2], Paolo Pinton [1,2,3,*],and Barbara Zavan [3,5,*]

Supplementary figures

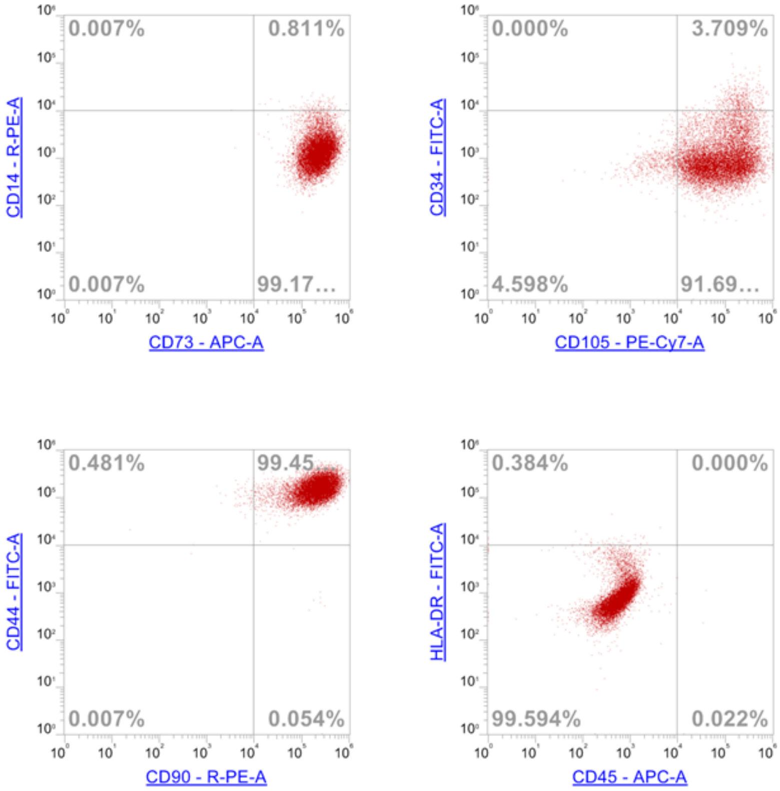

Figure 1. Detection of cell surface markers by flow cytometry. Adipose derived-cells show positive expression to CD44, CD73, CD90, and CD105, and negative to CD14, CD34, CD45, and HLA-DR surface markers.

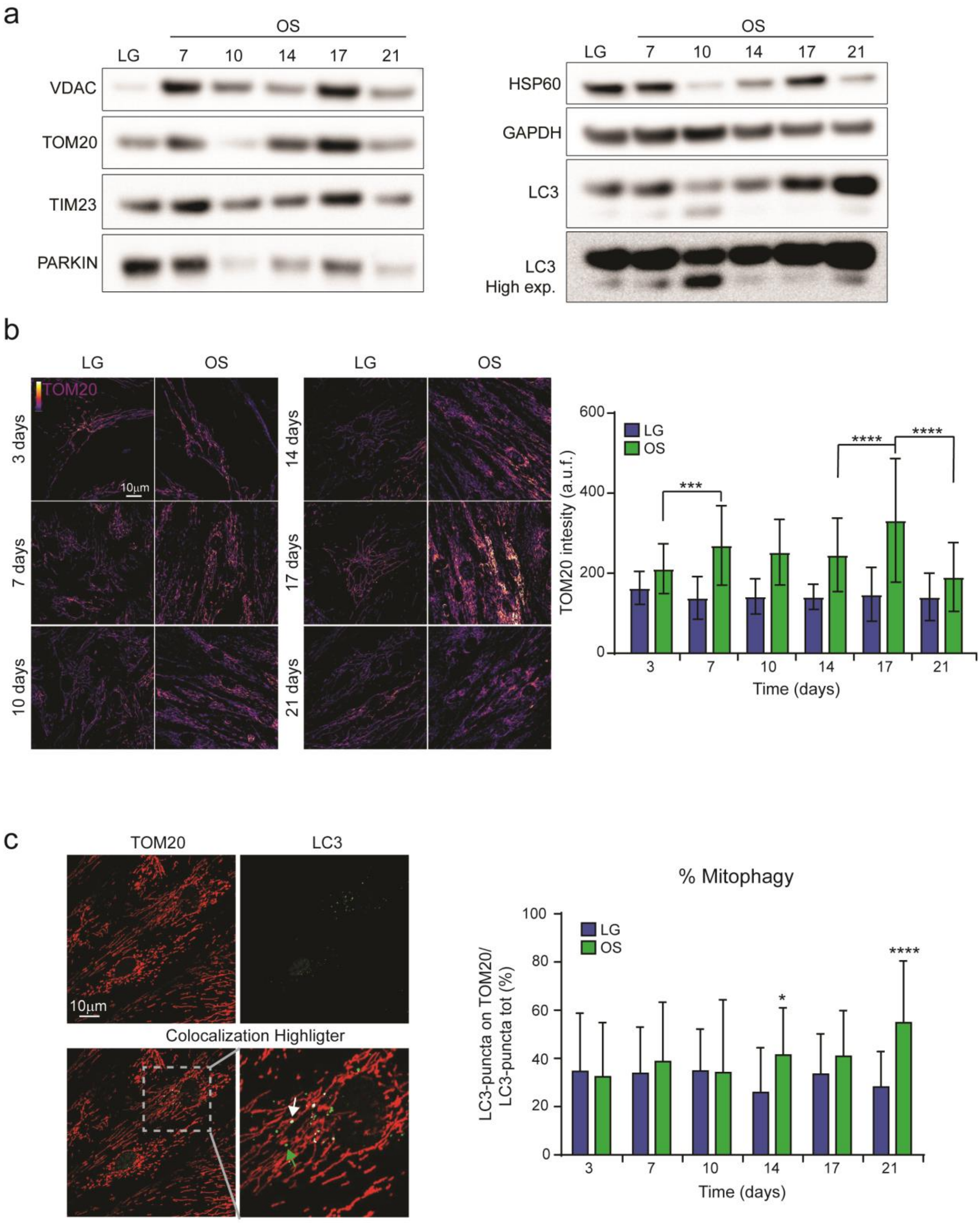

Figure 2. Mitophagy activation in the late stage of osteogenesis. MSCs were cultured in LG and osteogenic (OS) media for 3, 7, 10, 14, 17 and 21 days. (a) Representative immunoblots of HSP60, TOM20, VDAC, TIM23, PARKIN, LC3 and GAPDH protein levels. (b) Representative images and quantification of the anti-TOM20 Ab detected by immunofluorescence. (c) Representative images and quantification of mitophagic levels detected by colocalization of LC3 puncta and mitochondrial staining (TOM20). Magnifications 40X. Scale bar 10 μm. Data are derived from ≥ 3 independent experiments and are shown as the mean ± SD. ANOVA-test, ** $p<0.01$, *** $p<0.001$ with respect to the LG condition at the same time point, except where otherwise indicated.

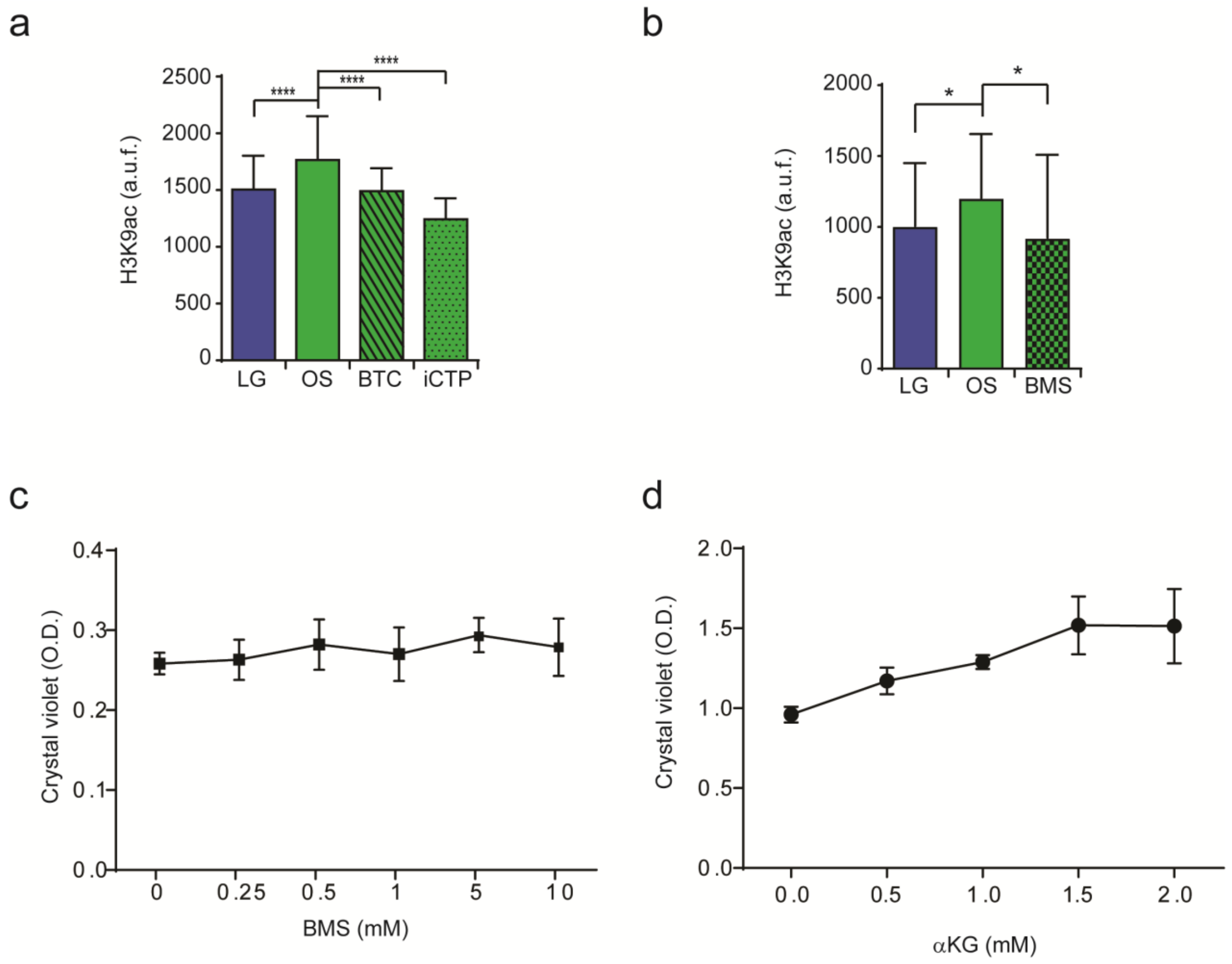

Figure 3. Modulation of citrate transport system. (a, b) Quantification of H3K9me3 in MSCs cultured in LG and OS media; where indicated, BTC [5 mM], iCTP [500] μM and BMS [1 mM] were added for 21 days. Data are derived from ≥ 3 independent experiments and are shown as the mean ± SD. ANOVA-test, * $p<0.05$, **** $p<0.0001$. (c, d) Cell survival assay to determine the concentration of BMS (c) and αKG (d).
